# The Surprisingly Positive Effect of Zinc-Phthalocyanines With High Photodynamic Therapy Efficacy of Melanoma Cancer

**DOI:** 10.3389/fchem.2022.825716

**Published:** 2022-03-14

**Authors:** Kelly A. D. F. Castro, Juliana A. Prandini, Juliana Cristina Biazzotto, João P. C. Tomé, Roberto S. da Silva, Leandro M. O. Lourenço

**Affiliations:** ^1^ Department of Biomolecular Sciences, Faculty of Pharmaceutical Sciences of Ribeirão Preto, University of São Paulo, Ribeirão Preto, Brazil; ^2^ Centro de Química Estrutural, Institute of Molecular Sciences & Departamento de Engenharia Química, Instituto Superior Técnico, Universidade de Lisboa, Lisboa, Portugal; ^3^ LAQV-REQUIMTE, Chemistry Department, University of Aveiro, Aveiro, Portugal

**Keywords:** photodynamic therapy (PDT), photosensitizer, cationic phthalocyanine, melanoma, cancer cells, *in vitro* assay

## Abstract

Phthalocyanine (Pc) dyes are photoactive molecules that can absorb and emit light in the visible spectrum, especially in the red region of the spectrum, with great potential for biological scopes. For this target, it is important to guarantee a high Pc solubility, and the use of suitable pyridinium units on their structure can be a good strategy to use effective photosensitizers (PSs) for photodynamic therapy (PDT) against cancer cells. Zn(II) phthalocyanines (ZnPcs) conjugated with thiopyridinium units (1–3) were evaluated as PS drugs against B16F10 melanoma cells, and their photophysical, photochemical, and *in vitro* photobiological properties were determined. The photodynamic efficiency of the tetra- and octa-cationic ZnPcs 1–3 was studied and compared at 1, 2, 5, 10, and 20 µM. The different number of charge units, and the presence/absence of *a*-F atoms on the Pc structure, contributes for their PDT efficacy. The 3-(4′,5′-dimethylthiazol-2′-yl)-2,5-diphenyl tetrazolium bromide (MTT) assays on B16F10 melanoma cells show a moderate to high capacity to be photoinactivated by ZnPcs 1–3 (ZnPc 1 > ZnPc 2 > ZnPc 3). The best PDT conditions were found at a Pc concentration of 20 μM, under red light (*λ* = 660 ± 20 nm) at an irradiance of 4.5 mW/cm^2^ for 667 s (light dose of 3 J/cm^2^). In these conditions, it is noteworthy that the cationic ZnPc **1** shows a promising photoinactivation ratio, reaching the detection limit of the MTT method. Moreover, these results are comparable to the better ones in the literature.

## Introduction

Melanoma, a well-known malignant, aggressive, and invasive skin carcinoma, is formed by a decontrolled transformation of melanocytes, which is the main reason of cell death in skin cancer (Siegel et al., 2021). From the global scientific data of the International Agency for Research on Cancer (IARC) of the World Health Organization (WHO), the occurrence of melanoma health problems has been growing over time and represents a high ratio of skin cancer deaths (Globocan. Melanoma of ski, 2020). Recently, the Global Cancer Statistics reported the possible occurrence of 324,635 melanoma cases of skin cancer and estimated 57,043 cancer fatalities to have happened in 185 countries during 2020 (Sung et al., 2021). It is noteworthy that more than half of the melanomas evidence BRAF V600 mutations (Ascierto et al., 2012) with ∼25% of neuroblastoma RAS viral oncogene homolog (NRAS) gene mutations (Rajkumar and Watson, 2016). For the clinical treatment, surgery and chemotherapy processes are active and useful options for patients diagnosed with melanoma disease. For growing-phase melanoma cases, medicinal treatments have been remarkably developed with the incorporation of immune inhibitor types and selective therapy such as BRAF and mitogen-activated protein kinase (MEK) inhibitors and programmed cell death ligand-1 (PDL-1) blockage (Ribas et al., 2020). However, the use of these components has limitations due to the high rate of innate or developed resistance in progressive metastatic melanoma (Welsh et al., 2016; Kozar et al., 2019; Vasan et al., 2019; Savoia et al., 2020).

Immunotherapy and other cancer therapies have been explored with relative success, increasing the survival of patients with malignant melanoma disease. However, the response in some patients is not effective due to the development of melanoma resistance after treatment (Winder and Virós, 2018; Czarnecka et al., 2020). Indeed, to combat this intrinsic resistance to the existing methodologies of treatment, photodynamic therapy (PDT) is a therapeutic alternative that can be used as an encouraging clinical approach (Marciel et al., 2017; van Straten et al., 2017; Valli et al., 2019). The PDT process involves the biological administration of a photosensitizer (PS) drug that is activated by suitable visible or near-infrared lights, which in the presence of cellular molecular oxygen generates highly cytotoxic reactive oxygen species (ROS), including the singlet oxygen (^1^O_2_) (Karunakaran et al., 2013; Lourenço et al., 2014a; Saenz et al., 2017; Pereira et al., 2018; Zhang et al., 2020; Castro et al., 2021). These ROS are responsible for the reduction or extinction of the targeted cancer cells or tumor tissues (Lourenço et al., 2014b; Ferreira et al., 2020).

Phthalocyanine (Pc) derivatives are photoactive compounds (ideally absorb light in the UV–Vis spectrum), especially in the red to near-infrared regions, which allow a higher tissue penetration and, consequently, a better PDT response (Camerin et al., 2010; Vummidi et al., 2013; Lo et al., 2020; Lopes-Nunes et al., 2020; Galstyan, 2021; Janas et al., 2021; Ribeiro and Lourenço, 2021). However, Pc macrocycles have weak solubility in various organic solvents or in aqueous media, and therefore, to minimize this drawback, it is essential to incorporate “bio”motifs, such as biologics or charged groups, including cationic pyridinium units on their structure to improve their amphiphilicity (Lourenço et al., 2015; Lourenço et al., 2019; Gamelas et al., 2020; Lopes-Nunes et al., 2020; Pereira et al., 2020; Revuelta-Maza et al., 2020; Revuelta‐Maza et al., 2021). Different peripheral substituents (α- and/or *ß*-positions) and their number usually give distinct photophysical, photochemical, and PDT results (Halaskova et al., 2021).

Recently, Valli et al. (2020) described the oxidative stress-induced apoptotic and autophagic signaling pathways using ZnPc dyes for PDT of melanoma cells, the induction process of apoptotic response, and a triggering protective autophagy. The same authors also reported the oxidative stress caused by light irradiation of ZnPcs inducing a dual apoptotic and necrotic response in melanoma cells. Mantareva et al. (2005) reported long-wavelength-absorbing cationic ZnPcs as fluorescent contrast agents for B16 pigmented melanoma. In their work, the Pcs were found to selectively accumulate in the target tumor, providing a potential application for fluorescence detection in clinical practice. Zheng et al. (2020) developed non-aggregated ZnPcs with hexadeca cations for antitumor and antibacterial PDT, evidencing that the quaternized compounds are efficient and can be used as promising PS drugs.

**FIGURE 1:**
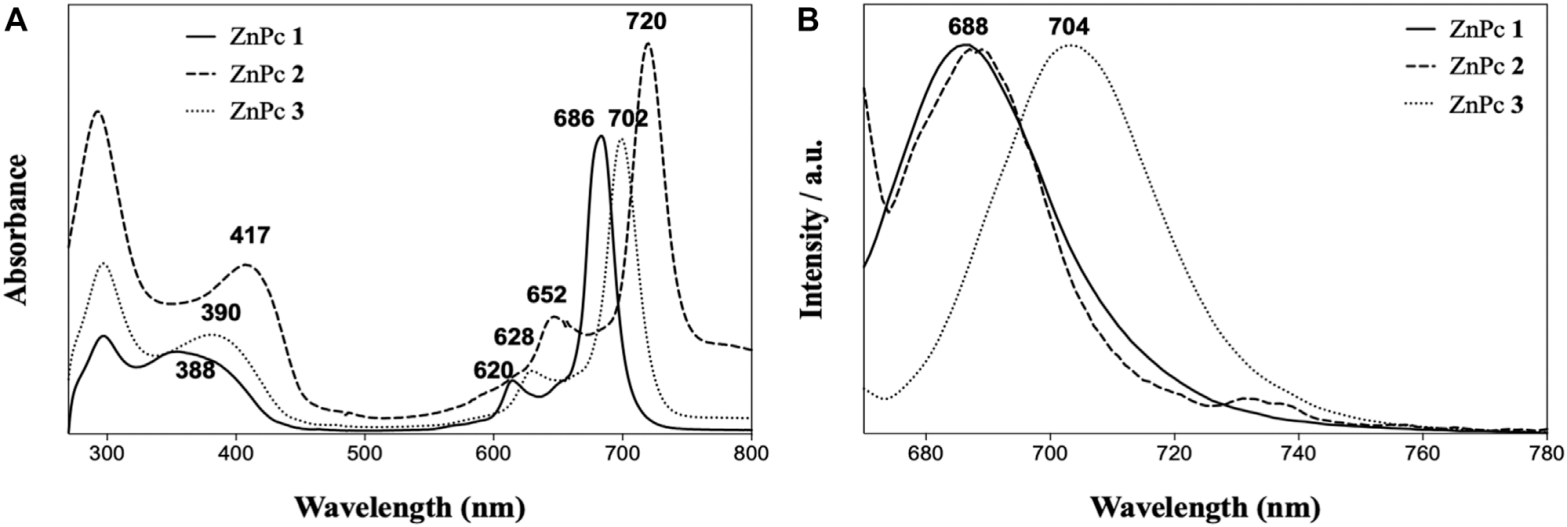
Spectra of **(A)** UV–Vis absorption and **(B)** normalized emission (λ_exc._ = 660 nm) of ZnPcs 1–3 in DMF.

The worldwide interest in cancer treatment, such as the very aggressive melanoma, led us to study a series of three thiopyridinium ZnPc dyes 1–3 (Figure 1), previously synthetized (Pereira et al., 2012) towards the photoinactivation of B16F10 melanoma cells.

## Experimental

All reagents were purchased from Sigma-Aldrich, Merck, or Gibco (without further purification). Analytical TLC was carried out on pre-coated silica gel sheets (Merck, 60, 0.2 mm). ^1^H, ^19^F, and ^13^C NMR spectra of Zn(II) phthalocyanine dyes (ZnPcs **1**–**3**) (Pereira et al., 2012) were recorded on a Bruker Avance-300 spectrometer at 300.13, 282.38, and 75.47 MHz, respectively, or on a Bruker Avance-500 (^13^C NMR at 125.77 MHz). The characterization of the obtained compounds corresponded to their full characterization previously reported (Pereira et al., 2012). Absorption and fluorescence spectra were recorded using an Agilent 8,453 and F4500 Hitachi spectrofluorometer (λ_exc._ at 660 nm, emission range 670–800 nm), respectively. The fluorescence emission spectra of Pc derivatives (C = 1 × 10^−6^ M) were recorded in DMF in 1 × 1 cm quartz optical cells under normal atmospheric conditions on a computer-controlled F4500 Hitachi spectrofluorometer. The fluorescence quantum yields were obtained using the commercial Zn(II) phthalocyanine (ZnPc) as standard (Gümrükçü et al., 2014) at optical density (O.D.) = 0.05 and excitation wavelength at 660 nm. The widths of both excitation and emission slits were set at 2.0 nm. The singlet oxygen quantum yield (Ф_Δ_) generated by the ZnPc dyes (ZnPcs 1–3) were determined from the rate of decay of the ^1^O_2_ phosphorescence at 1,270 nm using an Edinburgh F900 instrument (Edinburgh, UK) consisting of a Rainbow OPO (Quantel Laser, France), 10 Hz, 2 mJ/pulse, which was pumped by a Brilliant NdYAG laser (Quantel Laser, France), and using ZnPc as the standard in DMF. The absorbance of the sample was determined in DMF and adjusted at O.D. = 0.1 with excitation wavelength at 660 nm. The 3-(4′,5′-dimethylthiazol-2′-yl)-2,5-diphenyl tetrazolium bromide (MTT) assays were performed to test the *in vitro* cytotoxicity of Zn(II) phthalocyanines (ZnPcs 1–3) against B16F10 melanoma cells.

### Synthesis of Thiopyridinium Zn(II) Phthalocyanines

The cationic ZnPcs1–3 (Figure 1) were obtained from the experimental procedure reported in the literature (Pereira et al., 2012). Synthesis of the thiopyridyl ZnPcs was achieved either from a tetramerization process of phthalonitrile derivatives or *via* post-modification of the commercial Zn(II) hexadecafluorphthalocyanine (ZnPcF_16_). Then, the obtained thiopyridyl ZnPcs were cationized using methyl iodide to obtain the quaternized ZnPcs 1–3.

### Cell Culture

B16F10 cells (murine melanoma cell line) were purchased from the American Type Culture Collection (ATCC®, N° CRL-6324TM) and grown in Roswell Park Memorial Institute (RPMI)-1,640 culture medium supplemented with 10% fetal bovine serum (FBS), 100 units/mL of penicillin, and 10 mg/ml of streptomycin.

### Photobiological Studies *In Vitro*

An amount of 2 × 10^4^ cells/well were seeded into 96-well plates and, subsequently, incubated for 24 h at 37°C in a 5% CO_2_ humidified atmosphere. After the incubation process, the cultures were incubated with different concentrations of ZnPcs 1–3 (1, 2, 5, 10, and 20 μM) for 4 h. The cultures were gently rinsed with phosphate buffered saline (PBS) solution, Roswell Park Memorial Institute (RPMI)-1,640 medium without phenol red was added, and then, PDT assays were performed. The cells were exposed to red light (emission peak maximum at 660 nm) emitted by an array of 96 light-emitting diodes (LEDs). The light irradiation dose was 3 J/cm^2^. After irradiation, the cultures were maintained at 37°C in a 5% CO_2_ humidified atmosphere. After 24 h of incubation, the cell viability was evaluated using MTT assay. Dark assays were performed at the same conditions used in the PDT studies. The irradiated control, cells without Pcs, was also evaluated. Three independent assays were performed with six replicates.

### Photobleaching Studies

The photobleaching studies of ZnPcs 1–3 were evaluated by exposing a solution of each ZnPc in RPMI-1640 (10 μM) under the same conditions used in the PDT experiments. The stability of ZnPcs 1–3 was verified by analyzing the absorption spectra at regular intervals for up to 10 min.

### Cellular Uptake

An amount of 5 × 10^4^ cells/well were seeded into 24-well plates and, subsequently, incubated for 24 h under the same conditions used in the photosensitization experiments. After this time, the supernatant was removed, and the cells were incubated with each PS at 20 μM for 4 h. Subsequently, the cells were washed twice with PBS, and 500 μL of serum-free RPMI without phenol red was added. The cells were observed by fluorescence microscopy (Nikon Eclipse Ti Microscope model TI-FL). Filter Cy5 (λ_exc._ = 620/660 nm and λ_em._ = 662.5 to 737.5 nm) was used for ZnPc detection.

### Statistical Analysis

The statistical analysis was performed by an unvaried ANOVA using GraphPrism 7. Similarity of variance was assumed with Bonferroni’s post hoc test for pairwise comparisons. Results with *p* ≤ 0.05 were considered statistically significant.

## Results and Discussion

### Synthesis of Thiopyridinium Zn(II) Phthalocyanine Dyes

**SCHEME 6:**
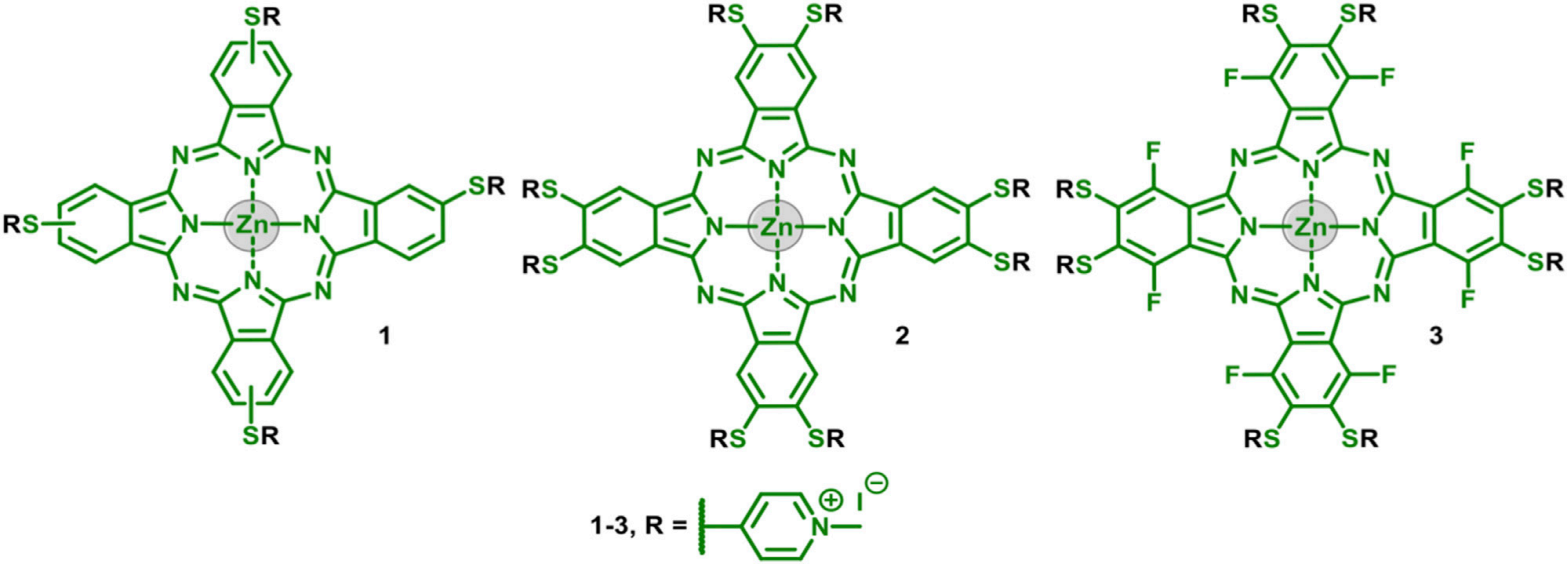
Chemical structures of cationic ZnPcs 1–3.

The synthesis and characterization of phthalocyanines ZnPcs 1–3 containing thiopyridinium substituents (Scheme 6) was performed. The compounds were fully characterized by NMR, UV–Vis absorption, and emission spectroscopy, as well as mass spectrometry, as previously described in the literature (Pereira et al., 2012).

From the prepared compounds, it is important to highlight that the peripheral moieties not only influenced the photochemical and photophysical properties but also the photostability, production of singlet oxygen, lipophilicity, and cellular uptake. The presence of fluorine atoms (in ZnPc 3) decreases the potency of this compound against B16F10 cancer cells due to its low cellular uptake and ability to generate ^1^O_2_ when compared to ZnPc 1 and ZnPc 2 (vide infra) (Shah and Westwell, 2007; Goslinski and Piskorz, 2011).

### Photophysical Properties of Thiopyridinium Zn(II) Phthalocyanines

The photophysical properties depend on the nature and number of the peripheral substituents and in this case, by the presence/absence of *a*-F atoms in the Pc structure. The UV–Vis spectra of ZnPcs 1–3 showed absorption Soret bands at 388, 390, and 417 nm, respectively, and Q-band maxima from 686 to 720 nm in DMF solutions (Figure 1; Table 1), which is attributed, respectively, to the S_0_ → S_2_ and S_0_ → S_1_ transitions that are characteristic for phthalocyanines in the monomeric form. For the emission spectra of ZnPcs 1–3, obtained after excitation at λ = 660 nm, it was observed that the typical bands centered at 688 nm for ZnPcs 1 and 2 and 704 nm for ZnPc 3 relative to the S_0_ → S_1_ transitions. For ZnPc 2, two additional bands at *ca.* 730 nm were observed, most probably associated to the vibrational state transitions.

**TABLE 1:** Photophysical properties of compounds ZnPcs 1–3 in DMF solutions.

ZnPcs	Soret band absorption	Q-band absorption	Emission	Ф_F_^a,b^	Ф_Δ_^c^
	λ_max._ (nm)	λ_max._ (nm)	λ_max._ (nm)		
**1**	388	620/686	688	0.23	0.67
**2**	390	628/702	704	0.18	0.17
**3**	417	652/720	688	0.12	0.07

a From reference (Wibmer et al., 2015).

b Using ZnPc as reference in DMF (Ф_F_: 0.28), O.D.: 0.05 at λ_exc._ of 660 nm.

c Using ZnPc as reference in DMF (Ф_Δ_: 0.56), O.D.: 0.1 at λ_exc._ of 660 nm.

It is noteworthy that the compound ZnPc 1 was the one that showed a higher fluorescence quantum yield (Φ_F_ = 0.23), followed by ZnPc 2 and ZnPc 3 (Table 1). Comparing the Ф_F_ values of ZnPc 3 and ZnPc, the reduction of Ф_F_ value for ZnPc 3 can be explained partially by the electron-withdrawing effects of the F atoms on their structure that increase the radiative decay rates. The number of thiopyridinium groups also influenced the Ф_F_ values: ZnPc 1 > ZnPc 2 > ZnPc 3.

Additionally, the photobleaching studies under the same conditions of the biological experiments were carried out. Following the UV–Vis analysis, the decrease in the characteristic phthalocyanine absorption band as a function of time upon exposure to irradiation was relatively small (data not shown), suggesting remarkable photostability for all compounds under red light irradiation as previously reported by some of us (Pereira et al., 2012), making the compounds attractive for PDT studies. Moreover, the cationic compounds of ZnPcs 1 and 2 evidence high solubility in water media due to the presence of four or eight positive charges. However, the ZnPc 3 with *a*-F on their macrocycle showed less solubility.

**FIGURE 2:**
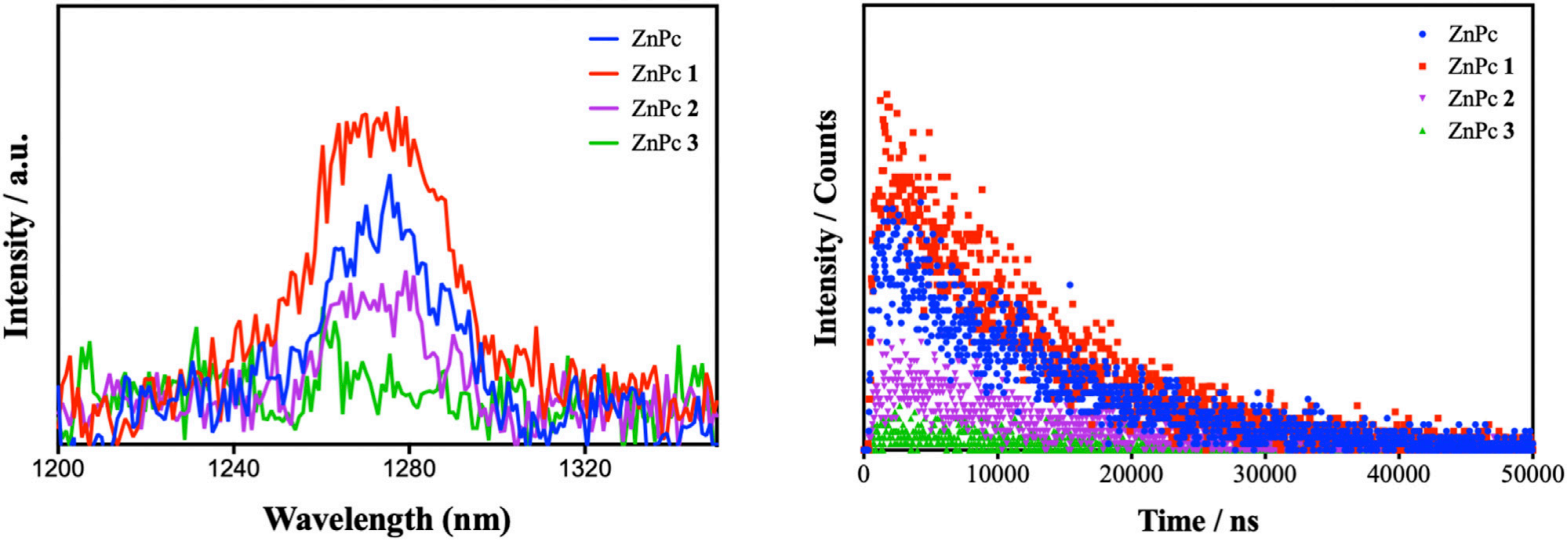
**(A)** Emission spectra resolved direct detection of ^1^O_2_ species and **(B)** time-resolved direct detection of ^1^O_2_ species generated by ZnPc reference and ZnPcs 1–3.

Upon light irradiation at an excitation wavelength of 660 nm, the ZnPcs in their excited state interact with molecular oxygen to generate ROS, especially ^1^O_2_ species. As can be seen in Figure 2, the production of ^1^O_2_ species was performed by monitoring their fluorescence emission at 1,270 nm. Singlet oxygen quantum yields (Ф_Δ_) of ZnPcs 1–3 were determined and compared to the ZnPc as a standard reference, where a significant increase for the ^1^O_2_ generation for ZnPc 1 was observed. Moreover, the Φ_Δ_ values of ZnPcs 1–3 followed the descending order, ZnPc 1 > ZnPc 2 > ZnPc 3 (Table 1), which are correlated with the number of thiopyridinium groups and presence/absence of *a*-F atoms (heavy atom effect) on the phthalocyanine backbone. In fact, the photophysical properties of the studied phthalocyanines could be affected by various molecular aspects, including extended π conjugation, structural distortion, and internal heavy atom. As expected, the fluorescence is quenched as shown in Table 1 due to the heavy atom effect of F atoms. On the other hand, the lowest ФD observed for ZnPc 3 maybe due to the combination of other effects resulting in low spin orbital coupling with consequent decreases in the triplet quantum yield. Since the triplet state decays at a relatively faster rate, the efficiency of oxygen quenching of the triplet state decreases. In addition, due to its slightly lower solubility compared to ZnPc 1 and ZnPc 2 because of interactions, fluor–fluor and fluor–hydrogen cannot be ruled out (Murray et al., 2021).

### Photobiological Studies With B16F10 Melanoma Cells

**FIGURE 3:**
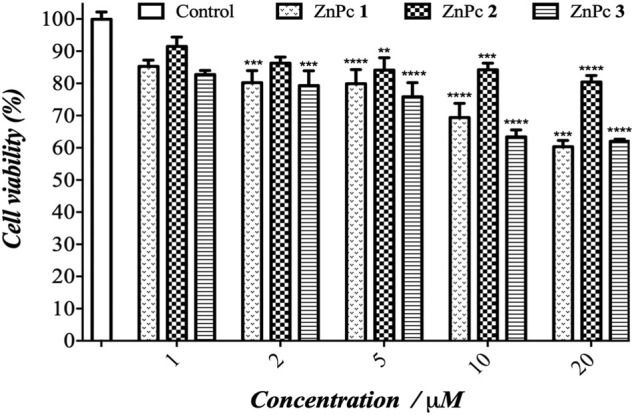
Dark cytotoxicity of ZnPcs 1–3 in B16F10 cells as a function of PS concentration. The results are presented as mean ± standard deviation. Statistical significance: **p < 0.01, ***p < 0.001, and ****p < 0.0001 *vs.* control.

Initially, the cytotoxicity studies of ZnPcs 1–3 were performed through cell viability of murine melanoma cells. The results are compiled in Figure 3 and show that the cellular viability is reduced ∼20–35% at higher concentrations (10 and 20 μM). These results suggest that ZnPcs 1–3 have a slight dark toxicity effect according to the concentration administered. However, the cytotoxic effect is slightly reduced at lower concentrations of 1 and 2 μM, and the cell viability is higher than 85%. Nevertheless, it was reported that the presence of 4-aminopyridine as the axial ligand in Ru(II) phthalocyanines induces a relative cytotoxicity effect against B16F10 cells due to their interaction with cell components (Martins et al., 2020). The cell viability decrease was significant at 1 μM (reduction in the cell viability = 62.6% compared to control); however, our results indicated an opposite effect because we observed a non-significant effect on this concentration.

**FIGURE 4:**
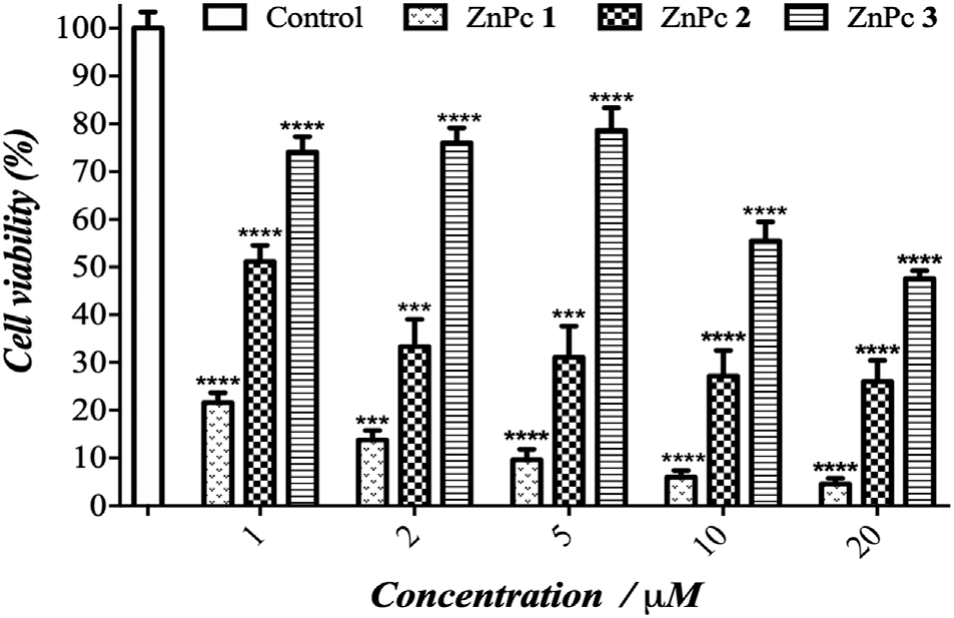
Photocytotoxicity of ZnPcs 1–3 against B16F10 cells as a function of PS concentration under red light and applying a light dose of 3 J/cm^2^. The results are presented as mean ± standard deviation. Statistical significance: ***p < 0.001 and ****p < 0.0001 *vs.* control. The control cells without PS were irradiated with the same conditions used in the PDT studies.

The PDT effect of ZnPcs 1–3 is clearly evidenced in melanoma cells upon exposure to a light dose of 3 J/cm^2^ of red light (Figure 4). The cell viability is significantly reduced and indicates the occurrence of cell death. The PDT efficiency can be directly correlated to the singlet oxygen production, following the best performance order of ZnPc 1 > ZnPc **2** > ZnPc 3. Despite the presence of fluorine atoms increasing the lipophilicity of ZnPc 3, this PS showed the lowest Ф_Δ_ value. ZnPc 1 showed 3.5 to 10 times higher (photo)toxicity compared to ZnPc 3 under the same light conditions. The results of phototoxicity on B16F10 melanoma cells suggest that phthalocyanine phototoxic effects are also PS dose dependent. Notably, the ZnPc 1 caused a significant PDT response at lower concentrations (1–20 µM), showing a considerable viability reduction at 1 µM (79%), and reached the detection limit of the MTT method at the highest concentrations used (10–20 µM). The concentration dependence is evident for ZnPc 1 with the increase in cell death of 79.4–94.4% at a concentration of 1–20 μM at the light dose of 3 J/cm^2^, respectively. For ZnPc **3** (lowest Ф_Δ_), the dependence on concentration is even more evident and the cell death was observed at 1 and 20 µM with a ratio of 26 and 53%, respectively.

In fact, the obtained PDT results are surprisingly comparable to the ones with other photosensitizers reported in the literature (Woodburn et al., 1998; Sparsa et al., 2013; Ma et al., 2015; Aishwarya and Sanjay, 2018), especially with some of them clinically approved for various types of cancer by the Food and Drug Administration (e.g., Photofrin or 5-aminolevulinic acid (5-ALA)), so we conclude that the values are comparable with those reported (Woodburn et al., 1998; Sparsa et al., 2013; Ma et al., 2015; Aishwarya and Sanjay, 2018). In addition, it was observed that the remarkable cell viability reduction induced by ROS was as expected. Currently, some studies indicate that such kind of pigmented melanomas are unresponsive to PDT with Photofrin because of melanin interference. In turn, if we talk about how other therapeutic approaches are used with, e.g., cisplatin drugs, the viability reduction effect is higher compared to those described by us, however, with high secondary effects.

The photocytotoxicity of (5-ALA)-induced protoporphyrin IX (PpIX) accumulation against B16F10 cells varied as a function of PS concentration (light dose = 37 J/cm^2^, concentrations of 5-ALA of 0–20 mM) (Sparsa et al., 2013). Silva and co-workers showed that the complex *cis*-[Ru(H-dcbpy-)_2_(Cl) (NO)][Na_4_(Tb(TsPc) (acac)] (*cis*-[RuPc]) at 0.5 mM exhibited some dark toxicity (*ca*. 20%). However, when B16F10 cells were irradiated in the presence of the *cis*-[RuPc], the cell viability dropped significantly (*ca.* 80%). From our results in the B16F10 cells, it was observed that low doses of ZnPcs **1–3** are enough for PDT efficacy (Cicillini et al., 2009).

Recently, Silva and co-workers improved the PDT effectiveness through a combination with photobiomodulation (PBM) using ruthenium phthalocyanine (RuPc) as a PS drug. The use of PBM followed by the PDT approach has been previously described. The reactive oxygen and nitrogen species (RONS) and ROS production and cellular uptake justify the increased PDT efficiency. In these studies, low concentrations of RuPc were used (up 1.0 μM) against A375 melanoma cells at different light doses (1, 3, or 6 J/cm^2^) (Negri et al., 2019a). PDT assays were also performed for the B16F10 cells. The cell line was incubated for 24 h with 10 μM of RuPc, irradiated at 660 nm with a light dose of 8.9 J/cm^2^, and subsequently, resulted in 50% of cell death (Negri et al., 2019b). Nevertheless, it is worth mentioning that the *in vitro* results depend on the PS or cell line, incubation time, and other experimental conditions, such as the light dose in the case of PDT.

Tedesco and co-workers (Goto et al., 2017) reported the encapsulation of aluminium chloride phthalocyanine (ClAlPc) in solid lipid nanoparticles using the direct emulsification for PDT studies. Briefly, the authors noted a light dose dependency resulting in a cell viability decrease of 85% for the hybrid ClAlPc/SLN and 51% for the non-immobilized ClAlPc, both at 0.75 μg/ml and a light dose of 2 J/cm^2^.

**FIGURE 5:**
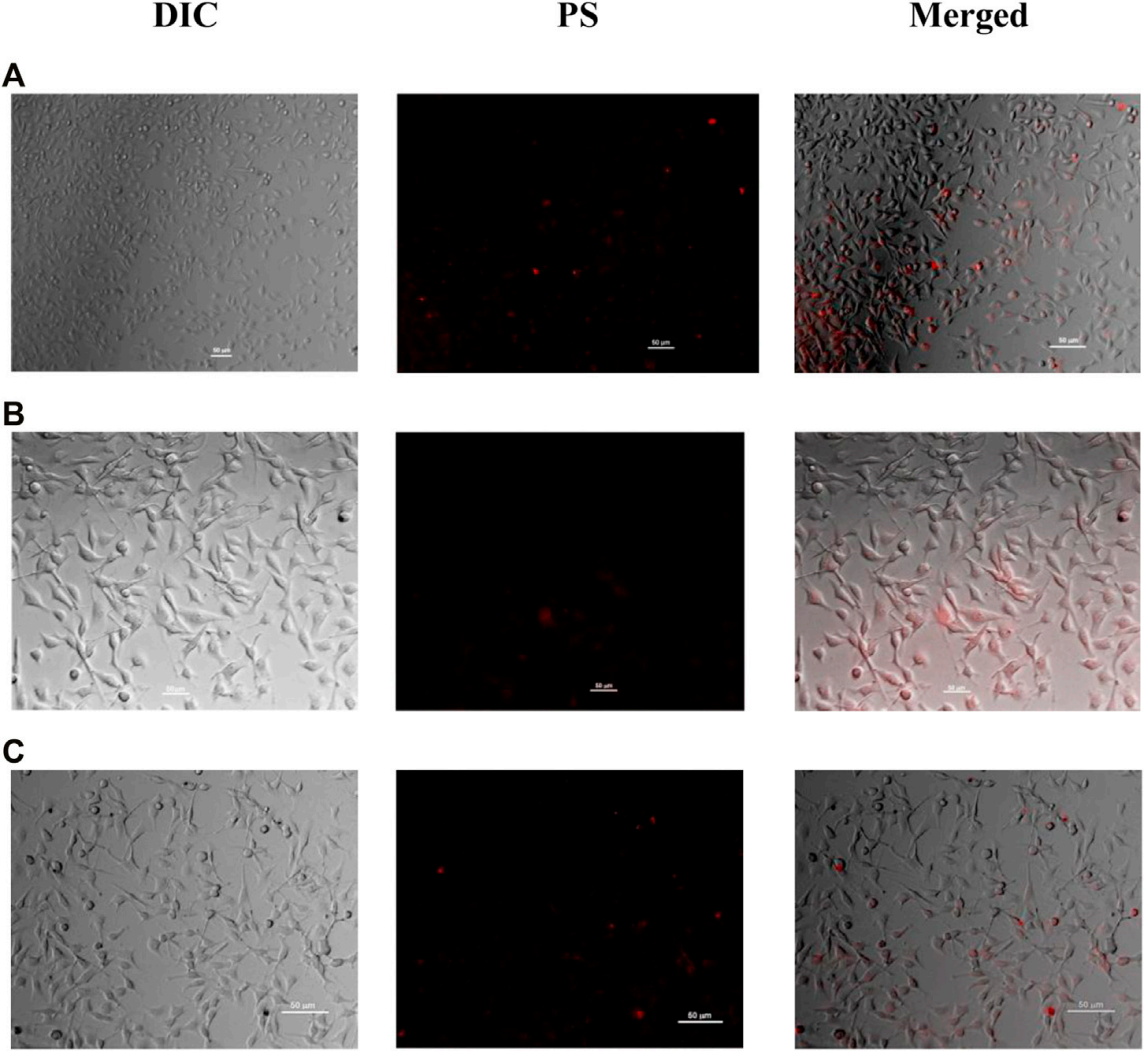
From left to right: differential interference contrast (DIC) and fluorescence microscopy images of B16F10 cells treated with **(A)** ZnPc **1**, **(B)** ZnPc **2**, and **(C)** ZnPc **3**. The images show the distribution of the ZnPc dyes (10 μM) in B16F10 cells after incubation for 4 h.

Knowing that, the cytotoxic effect of a PS drug is also affected by the cellular internalization, where the fluorescence microscopy images of B16F10 cells treated with all the compounds were obtained (Figure 5). The cellular internalization of ZnPcs 1–3 was confirmed by fluorescence microscopy, and as shown in Figure 5, the characteristic red fluorescence of our compounds was observed. The overlay of fluorescence images with bright field showed that all compounds are distributed in the cells. In addition, the cellular uptake apparently increased according to the MTT assays: ZnPc 1 > ZnPc 3 > ZnPc 2. In fact, the PDT performance of PS increases with its cellular uptake, and the PS internalization varies according to its chemical and physical properties.

## Conclusion

The tetra- and octa-thiopyridinium ZnPcs 1–3 were evaluated as PS drugs against B16F10 melanoma cells. ZnPcs 1–3 were administered at different concentrations (1–20 μM) under dark and light conditions. The ZnPc **1** was able to photoinactivate B16F10 melanoma cells reaching the detection limit of the MTT method under red-light irradiation. In an attempt to justify the obtained results, the photophysical, photochemical, and *in vitro* photobiological properties were evaluated and correlated. The better performance observed for ZnPc 1 was directly correlated with its highest singlet oxygen production compared to the others: ZnPcs 2 and 3. The PDT results showed that the choice of the adequate phthalocyanine backbone modulates the photophysical and photochemical properties and, consequently, the performance of PDT. Moreover, the best PDT conditions were found for ZnPc 1 at a concentration of 20 µM, under red light (λ = 660 ± 20 nm) at an irradiance of 4.5 mW/cm^2^ for 667 s (light dose of 3 J/cm^2^). From the aforementioned data, the cationic ZnPc 1 can be considered an interesting PS agent to treat melanoma cancer cells, especially B16F10 ones.

## Data Availability

The raw data supporting the conclusion of this article will be made available by the authors, without undue reservation.
